# Exercise protocols: The gap between preclinical and clinical exercise oncology studies

**DOI:** 10.1016/j.metop.2022.100165

**Published:** 2022-01-22

**Authors:** Mahmoud Delphan, Neda Delfan, Daniel West, Maryam Delfan

**Affiliations:** a Department of Physical Education and Sport Sciences, Islamic Azad University, Tehran, Iran; b Department of Exercise Physiology, Faculty of Physical Education and Sport Sciences, University of Tehran, Tehran, Iran; c KITE Research, Toronto Rehabilitation Institute, University Health Network, Toronto, Canada; d Faculty of Kinesiology and Physical Education, University of Toronto, Toronto, Ontario, Canada; e Department of Exercise Physiology, Faculty of Physical Education and Sport Sciences, Alzahra University, Tehran, Iran

**Keywords:** Preclinical, Clinical, Exercise Oncology, Translational failure, Gap analysis, Exercise protocols, Cancer-induced complications

## Abstract

**Introduction:**

Preclinical studies provide foundational knowledge to develop new effective treatments for use in clinical practice. Similar to *clinical* exercise oncology studies, it is also important to monitor, identify and/or avoid cancer-induced complications in *preclinical* (e.g., murine) exercise oncology studies. This may help close the gap between preclinical and clinical exercise oncology studies. The aim of the present mini review is to provide insight into exercise protocol design in preclinical exercise oncology studies in order to close the preclinical-clinical gap. A secondary aim was to examine exercise-responsive outcomes in the preclinical versus clinical setting.

**Method:**

We reviewed animal studies in exercise oncology*.* A literature search was performed in PubMed/Medline and studies in English were screened.

**Results:**

We found that the majority of preclinical exercise protocols have not been at least tested clinically. We found some evidence that certain outcomes of preclinical studies (e.g., markers of cellular and molecular adaptation) that translate to clinical studies. However, this translation was dependent on the use, by investigators in their study design, of suitable and applicable preclinical exercise protocols.

**Conclusions:**

Cancer and its treatment-induced complications (e.g., fatigue, cardiac atrophy, cachexia, etc.) have largely been ignored in the exercise protocols of preclinical oncology studies. Preclinical exercise oncology studies should consider the limitations of human exercise oncology studies when conducting gap analysis for their study design to increase the probability that findings related to mechanistic adaptations in exercise oncology will be translatable to the clinical setting. By virtue of paying heed to patient compliance and adverse effects, clinical exercise oncology research teams must design relevant, feasible exercise protocols; researchers in preclinical exercise oncology should also take such factors into consideration in order to help bridge the gap between preclinical and clinical studies in exercise oncology.

## Highlights

• Preclinical research provides foundational knowledge for the development of human experimental and clinical studies; however, direct translation of preclinical findings has a low success rate.
• Exercise can provide potent wide-ranging beneficial effects for people with chronic diseases, including cancer.
• A minority of preclinical exercise oncology studies account for cancer-related factors in their experimental design.
• To lessen the preclinical–clinical gap in exercise oncology research, preclinical exercise oncology studies should consider using experimental designs that are based on locomotor activity and/or VO2peak in animals bearing cancer.

## 1 Context

### 1.1 Translating protocols from preclinical to clinical studies

Gap analysis is a process that identifies what aspects of human study need to be supported by future preclinical studies. This process may also include defining the preclinical strategy that will address the requirements of the target regulatory body. In reviewing available information, literature should be evaluated not only for ‘content’ to support the preclinical strategy but also scrutinized for factors/issues that may render the findings not applicable to the investigator's goals (e.g., technical or procedural issues) [1]. (This has parallels to conducting a systematic review— identifying risks of bias as well as the standardized overall effect size).

Though gap analysis is time consuming, thanks to the harmonization of different regulatory bodies [1], subsequent preclinical studies could be conducted at multiple locations (e.g., outsourcing globally) to expedite time lines and minimize costs. It is well known that the rate of translating *pre*clinical success into viable clinical applications is very low (∼5%), due to many factors including poor experimental design, animal models and poor reporting owing to having not been tested in an practical manner [2]. Whereas various mitigation strategies are ongoing [3], the gap between preclinical and clinical utility shows little sign of closing [4]. Similar to investigational new drug development, translating preclinical exercise oncology findings to the clinical setting presents many challenges; for example, translating exercise protocols from the human to the animal and vice versa [5,6]. In this mini-review, we aim to highlight gaps between human and animal studies in exercise oncology, and discuss the exercise protocol framework to improve the translation rate between preclinical and clinical studies. We examine preclinical exercise protocols, their translation to clinical studies, and if/how clinical studies take into account preclinical exercise training frameworks. Lastly, we discuss the importance of developing an ‘optimal’ exercise training framework for preclinical exercise oncology in order to improve translation success into the clinical exercise oncology setting.

### 1.2 Mechanisms in exercise adaptation: considering limitations of human studies in preclinical studies

Exercise training has a myriad of pleiotropic effects on numerous cells, tissues, and organs of the body in both healthy as well as diseased states [2,7]. Exercise adaptations are underpinned by numerous signaling pathways as well as local and distant feedback loops that give rise to a new homeostatic state. The end result is an increase in cellular, tissue and organ fitness leading to wide-ranging disease improvements and decreases in health problems [[8], [9], [10], [11], [12], [13]]. Therefore, there is considerable potential value in being able to appropriately apply preclinical exercise to disease states such as cancer. This includes developing relevant preclinical exercise models and protocols, including accounting for genetic factors [[14], [15], [16]], as well as mode, intensity, and duration, respectively. Combined, these factors will influence molecular and cellular adaptation [[8], [9], [10], [11],^17^], despite the presence of disease [10,13]. Despite its relatively low adoption in modern healthcare, exercise training ‘prescription’ offers considerable therapeutic potential [10,12,13] given that exercise training has direct impacts – from modifying cell function to inter-tissue crosstalk [12] – in chronic diseases including cancer [13,18]. Indeed, it is probably valuable to consider exercise prescription from two ‘lenses’— considering both clinical benefit as well as understanding prescription/adaptation at a molecular level [8,10,13].

Little data exists regarding how modifying exercise protocol parameters modifies the mechanisms that influence cancer survival, both preclinically and clinically. To evaluate whether the results of preclinical exercise oncology findings translate to clinical exercise oncology, protocols in preclinical study should be designed based on the framework of protocols considering the limitations of clinical studies.

Human and animal exercise activate a myriad of cellular pathways which contribute to remodeling and/or adaptation [[8], [9], [10], [11],^13^]; however, the extent to which given pathways yields anticancer benefits, and whether this is of the similar significance in animal and humans, is unclear. Nevertheless there is evidence of preclinical-clinical commonalit; for example, high intensity interval training appears to evoke similar potent benefits [[19], [20], [21], [22], [23]]. Understanding the exercise tolerance thresholds (especially clinically) together with exercise stimulus thresholds (to elicit a meaningful beneficial physiological response) will combine to establish suitable exercise protocols oncology studies. Further, *expected* adaptations (e.g., derived from healthy populations) need to be examined with actual adaptions that occur in the cancer/cancer therapy environment, taking into account both the altered biological (e.g., inflammatory) and physical (e.g., altered locomotor activity) environments.

### 1.3 Cancer and treatment-related fatigue: ignored factors in preclinical exercise protocol design

Fatigue is one of the main pervasive side effects of cancer [[24], [25], [26]]. It is also among the most common and challenging symptom during and after treatment [24,[26], [27], [28]]. The mechanistic pathways of cancer-induced fatigue in human and animals are poorly understood but include bio-behavioral factors (e.g. mood, depression, stress and sleep disturbance) [[29], [30], [31], [32]], hypothalamic-pituitary-adrenal axis [29,30,32], neuroinflammation [[33], [34], [35]] and muscle wasting [30,32,36]. Several studies demonstrate that cancer [22,29,36] and its chemotherapy treatment lead to reduced voluntary wheel running activity of mice [25,[37], [38], [39], [40], [41]] or running speed reduced by 20% at the target intensity [42]. Cancer-induced skeletal muscle dysfunction [43,44] and cardiac atrophy [44] reduce locomotor activity and exercise capacity. Collectively, the debilitating complications of cancer – direct and indirect – are important to consider when designing preclinical exercise oncology studies. Results derived from preclinical studies that impose excessive pressure on animals to perform exercise training – often more than their voluntary capacity – can yield misleading interpretations and expectations of translation to the human condition [6], Whereas exercise parameters in the clinical setting are likely to be of a milder intensity and geared toward doing no harm (in line with patient's self-selected/voluntary effort), there is growing evidence (a recent systematic review identified 12 studies [45], all within the past decade) of efficacy/feasibility of high intensity training [[46], [47], [48]].

### 1.4 Exercise intensity in preclinical exercise oncology studies

VO2_peak_ measurement not only is reported as the gold standard assessment of exercise capacity in patients, but is a strong independent predictor of the cancer patients mortality [49]. In a preclinical setting, serial measurements of VO_2max_ are suggested to regulate running speed/exercise intensity [43]. Whereas running pace can be used as an indirect measure of oxidative capacity measuring VO_2peak_ in rodents provides a more informative/standardizable readout of exercise intensity for exercise interventions as well as cancer-induced changes in locomotor activity in rodents. Indeed, a remarkable decline in indirect VO_2max_, running speed [42], and endurance exercise capacity [50] of rats with cancer throughout the study period revealed that cancer-bearing animals do not reach maximal VO_2max_ compared to healthy controls. Based on these results [42,43,50], applying valid and reliable experimental models is essential to examine – and standardize across labs – the impact on cellular and molecular mechanism of exercise training for prevention, treatment and rehabilitation of chronic diseases [43].

In addition to improving understanding of mechanisms, the ability to standardize exercise protocols (e.g., based on VO2) will advance our understanding of the impact of cancer-, chemo- and hormone-therapy on function and fatigue. Although exercise training is a safe therapy to mitigate cancer-related fatigue and improve exercise tolerance in cancer survivors [51,52], and in mice [53,54], it is essential to consider the capacity of exercise tolerance and locomotor activity. Increased running speed/intensity during a preclinical exercise oncology study without considering exercise capacity may overlook cancer effects and treatment-induced side effects such as fatigue and reduced locomotor activity.

A method to assess fatigue in the rodent is locomotor activity reduction [29]. Recently, Dougherty JP et al. used a treadmill test to determine fatigue-like behavior in mice undergoing chemotherapy [55]. Although this method needs to be verified by future studies, it may be a good method to determine the side effect of cancer-, chemo- or hormone therapy in preclinical exercise intervention studies. Additionally, we have used [19] a method to design a suitable preclinical exercise protocol based on Leandro CG et al. [56] and Hoydal MA et al. [43]. We measured VO_2peak_ of breast cancer-bearing mice indirectly prior to starting the study. To assess the indirect VO_2peak_, mice started running on the motorized treadmill at a speed of 6 m min^−1^. The speed was increased by 2 m min^−1^ every 3 min until the mice were unable to run and to maintain running speed on the treadmill. We kept this method weekly for 5 weeks (once every 6 days), and then every other week for 5 weeks (once every twelve days) by the end of the study. Indirect VO_2peak_ measurement was performed in interventional groups, followed by 2 days rest to monitor any potential changes in mice running ability resulting from cancer (and/or adaptation to exercise), and also to select exercise intensity for the subsequent week. During our study [19], we observed that running ability and exercise tolerance of breast cancer bearing mice were significantly reduced after the second week of tumor palpation. Further, we found that running capacity of cancer bearing mice was progressively reduced, week by week, to the end of the study [19].

This Other preclinical studies have used a similar VO2-based frameworks [[20], [21], [22], [23]]. Similar protocols were also used in diabetic [57,58] and also fatty liver animals [12], aiming to generate information with a higher probability of translation to clinical studies. Using a progressive exercise protocol, or a protocol with a stable intensity during preclinical exercise oncology studies without estimating exercise capacity before and throughout the study may lower the translation rate, and widen and deepen the gap with clinical practice. Increasing running speed progressively [59,60] or stabilizing speed and intensity during the study [[61], [62], [63], [64], [65], [66], [67], [68]] may not take into account cancer-related complications and/or cancer therapeutic treatment-induced fatigue, potential cardiac atrophy and skeletal muscle dysfunction. Hence, preclinical exercise oncology studies should consider the intervention framework approaches based on the feasibility and suitability in clinical exercise oncology studies. Taken together, preclinical studies that have voluntary wheel running or exercise protocols designed based on VO_2peak_) may have a better chance of successfully translated to clinical studies. Kumar 2011 has said “preclinical information is used to estimate an initial safe starting dose and dosing regimen for human trials” [1]. Thus, the analogue for exercise oncology, the exercise protocol, requires similar careful monitoring and ‘dosing’ in order to better translate preclinical findings.

### 1.5 Summary and future directions

Preclinical exercise oncology studies provide essential supporting scientific insight for clinical oncology trials. Nevertheless, a large number of preclinical exercise protocols have not been translated in clinical practice due to poor methodology and lack of the framework validation of protocols. Accordingly, we recommend, as a direction for future study, to researchers use a well-defined exercise protocol (including reporting speed, slope and VO2_peak_) prior to and during the study, such as those described by Hoydal et al. [43], Dougherty JP et al. [55] and Delphan et al. [19]. However, future research is needed to measure VO2peak directly in cancer bearing animal (e.g. mice and rats). Voluntary wheel running protocols are also viable exercise framework that may better account for the fatiguing aspects of cancer/therapy [22]. In this way, the effects of cancer/therapy on exercise tolerance and locomotor activity in cancer bearing animals can be standardized and the impact on mechanistic pathways quantified more reproducibly toward the ultimate goal of clinical translation. Researchers who have a good understanding of exercise protocol development in healthy and disease states, and in preclinical and clinical settings, will be best positioned to translate the potentially beneficial preclinical exercise-based effects to the clinical setting and, ultimately, cancer patient populations more broadly.

## CRediT authorship contribution statement

**Mahmoud Delphan:** conceptualized the manuscript. **Neda Delfan:** reviewed literature contributed to the intellectual discussion, and revised the manuscript. **Daniel West:** reviewed literature contributed to the intellectual discussion, and revised the manuscript. **Maryam Delfan:** reviewed literature contributed to the intellectual discussion, and revised the manuscript.

## Declaration of competing interest

The authors declare no conflict of interest.
